# *Moringa oleifera* gum based silver and zinc oxide nanoparticles: green synthesis, characterization and their antibacterial potential against MRSA

**DOI:** 10.1186/s40824-021-00219-5

**Published:** 2021-05-08

**Authors:** Muhammad Irfan, Hira Munir, Hammad Ismail

**Affiliations:** Department of Biochemistry and Molecular Biology, University of Gujrat, 50700 Gujrat, Pakistan

**Keywords:** Nanoparticles, Green synthesis, Moringa oleifera, MRSA

## Abstract

**Background:**

Herein, we first time used the gum *Moringa oleifera* as reducing and capping agent for successful synthesis of silver nitrate and zinc oxide nanoparticles(NPs) through green synthesis approach. This study was aimed to check antibacterial activities of synthesized NPs against multidrug resistant bacteria *methicillin-resistant Staphylococcus aureus* (MRSA).

**Methods:**

Aqueous solutions of AgNO_3_ and purified gum powder were mixed with 1:1 ratio, autoclaved at 120^o^C for 2 min. NPs pellet collected after centrifugation at 10,000 g for 20 min. ZnO NPs were prepared by mixing purified gum powder and metal salt with1:1 ratio, heated (70^o^C) and stirred at 100 rpm for 4 h followed by centrifugation at 10,000 *g* for 20 min. Pellet was washed and calcinated at 400^o^C for 4 h. Antibacterial potential against *E. coli, S. aureus* and *methicillin-resistant Staphylococcus aureus* (MRSA) was assessed by widely used Kirby-Bauer antibiotic susceptibility test.

**Results:**

Optical observation of colour change from transparent to dark and *UV-Visible* analysis confirmed the synthesis of NPs. Fourier transform infrared spectroscopy (FTIR) of prepared nonmaterial revealed the characteristic AgNPs and ZnO stretch vibrations at wave number of 523 cm^− 1^ and 471 cm^− 1^resectively. Crystalline nature of AgNPs and ZnO NPs was confirmed by x-ray diffraction pattern with clear sharp Peaks. Scanning electron microscopy (SEM) revealed good surface morphology of AgNPs and ZnO NPs with 50nm and 60nm size respectively. AgNPs and ZnO NPs exhibited excellent antibacterial activity against *E. coli* (with zone of inhibition of 21 ± 02mm and 22 ± 03mm) and *S.aureus* ( with zone of inhibition of 20 ± 03mm and 21 ± 02mm) while good activity was observed against “super bug” *methicillin-resistant Staphylococcus aureus* (MRSA) with 16 ± 03mm ad 17 ± 02mm zone if inhibitions respectively.

**Conclusions:**

This novel addition of Moringa Gum based nanoparticles will open new dimensions in the field of nanomedicine and pharmaceutics especially against MDR bacterial strains.

**Supplementary Information:**

The online version contains supplementary material available at 10.1186/s40824-021-00219-5.

## Background

Plants gums/ resins due to their properties of non-toxicity, non-polluting, sustainable, recyclable, low costs, eco-friendliness, wide spread availability, biodegradability and biocompatibility have bestowed them unique position in the field of pharmaceuticals, nanoparticles synthesis and food industry[1]. Natural polysaccharides/biopolymers based nanoparticles have a lot of advantages over similar synthetic entities. These plants polymers do double actions i.e. they act as stabilizing as well as reducing agents for metal ions while synthesizing nanoparticles [2].

*Moringa oleifera (Moringaceae)* is highly important plant from medicinally point of view and is distributed in tropics and subtropics regions of world. Its different parts contain variety of important phenols, amino acids, proteins, vitamins and *β-*carotene, [3]

Different parts of this plant has amazing medicinal properties like antiulcer, diuretic, antitumor, antipyretic, anti-inflammatory, antispasmodic antiepileptic, antihypertensive, anti-diabetic ,cholesterol lowering and antioxidant. This plant is being used in indigenous health system of south Asian region particularly[3].

Over the past few decades, gums from natural sources are widely studies by the researchers due to their valuable physicochemical qualities. During past few years, different researchers have reported synthesis of metal nanoparticles using various plant gum/resins through green synthesis approach such as *gum cashew*[4], *olibanum gum*[5], *gum karya*[6]. *Gum ghatti*[7]. *Gum neem*[8], *accasia arabica gum*[9] and *gum kondagogu*[10].

The purified gum from *Moringa oleifera* plant was found to contain number of flavanoids, proteins and carbohydrates such as d-xylose, l-arabinose, d-glucuronic acid, d-galactose,d-mannose, and l-rhamnose[11].

The bio-based ways for silver and zinc oxide NPs synthesis present four major advantages: [1] enhanced biocompatibility (capped with biomolecules such as sugars, proteins, or metabolites); [2] less toxicity [3] trouble-free production (from extracts of plants, bacteria or fungi and silver salt and [4] less cost.

Multidrug-resistant bacteria (MDRB) or “super bugs” are exceptionally treacherous and pose a serious threat to global health systems as they can survive an attack from drug. Adaptive living style of bacteria with antibiotics causes them to modify their genetic makeup to emerge resistance against known antibiotics and their combinations. The appearance of nanoparticles as new antimicrobial agents has boosted up the research for tackling these superbugs. As nanoparticles target the bacterial cell through multiple pathways, it becomes difficult for bacteria to escape from these magical agents.

Zinc oxide NPs have been prepared via leaf extract of *Abutilon indicum*[12], *Aloe barbadensis*[13], *Indigofera tinctoria*[14], *Melia azedarach*, fungus and bacteria[15]. Recently, ZnO NPs have been synthesized via green approach through leaf extract of *Sidium gujava* [16], seed extracts of *Nigella sativa*[17], root extract of *Daucus carota*, leaf extracts of *Monsonia burkeana* [18]and *Mangifera indica*[19], *fungus Halomonas elongate and Aspergillus terreus*[20]. Considering important characteristic of Moringa Oleifera plant and bio based metal nanoparticles, we developed Gum based novel NPs for their further uses in pharmaceuticals.

## Methods

### Chemicals

Crude Gum Moringa (GM) was purchased from local market of Gujrat city and purified by ethanol precipitation method. Lab grade AgNO_3_ (Sigma-Aldrich-204,390) and Zn (NO3)_2_. 6H_2_O (Sigma-Aldrich-96,482) was taken from Biochemistry lab, University of Gujrat, Pakistan. Bacterial strains were procured from central lab of University of Veterinary & Animal Sciences – UVAS Lahore, Pakistan.

### Preparation of silver and ZnO nanoparticles using gum Moringa oleifera

Silver NPs were prepared by following Venkatesham et al., 2014 [21] with slight modification. Purified gum moringa (0.5 gr) was dissolved in 100 mL of mili-Q water and AgNO_3_ (0.5 gr) was mixed in 100 mL of milo-Q water. Then, 5 mL from each solution was taken, mixed and kept in autoclave at 15 psi pressure at 120^◦^Cfor 2 min.

ZnO nanoparticles were prepared with little modification to Ogunyemi et al., 2021 [22]. The procedure carried out by mixing 1:1 ratio *(v/v)* of zinc nitrate hepta-hydrate and the Gum sample. Solution was heated (70^o^C) and stirred continuously at 100 rpm for 4 h followed by centrifugation at 10,000 *g* for 20 min. Nanoparticles pellet obtained, washed with distilled water by discarding the supernatants and calcinated at 400^o^C for 4 h.

## Results

### 1) Characterization of AgNPs and ZnO NPs synthesized through “green” protocol

#### a) Optical Observation

**Fig. 1:**
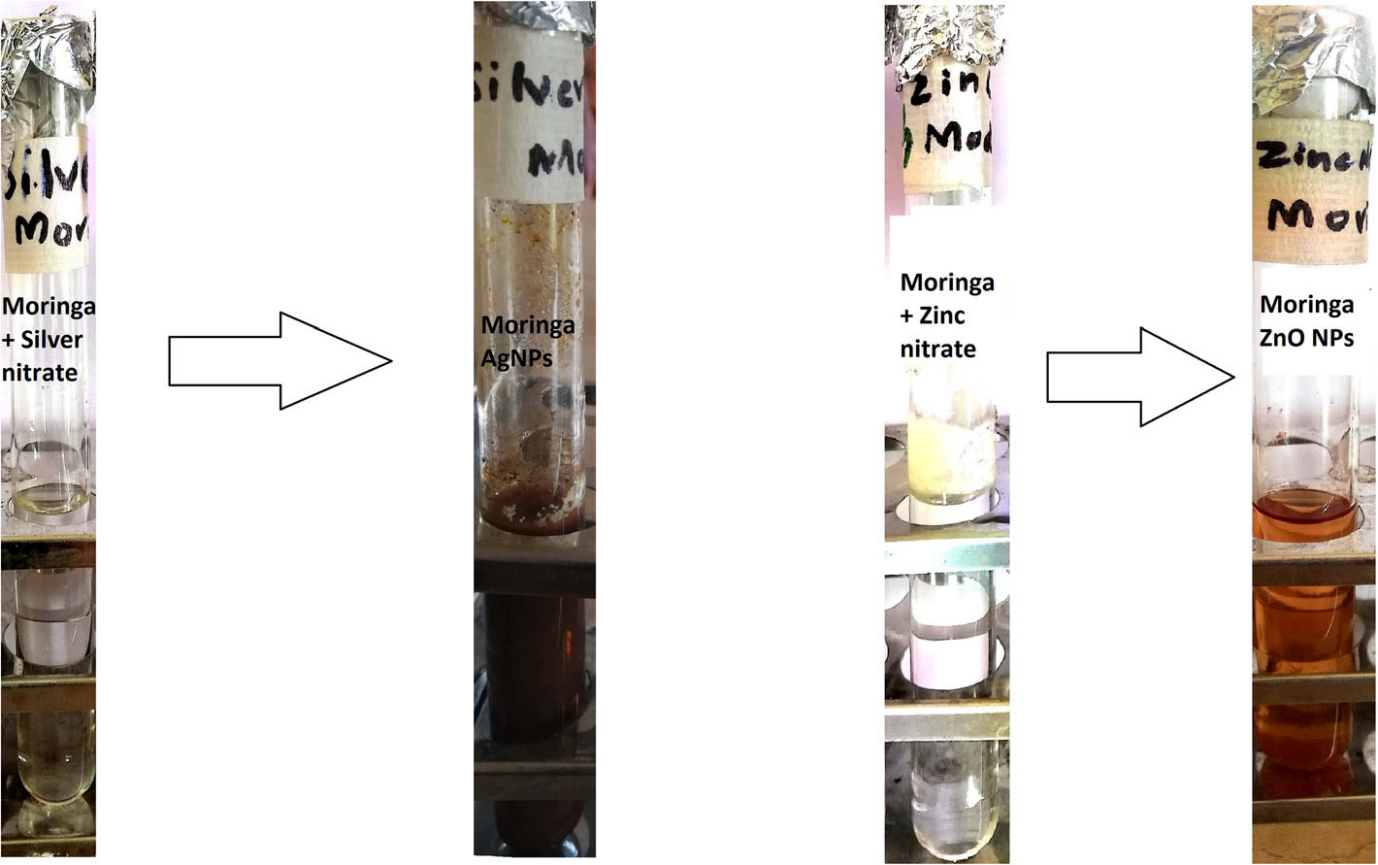
Colour Change from transparent to dark indicating the development of colloidal nanoparticles solution of AgNPs and ZnO NPs

Colour change of developed NPs solutions (Fig. 1) from transparent to dark brown and light brown (for Ag and ZnO NPs respectively) confirmed the formation of nanoparticles. These colours appeared because NPs reflect (scatter) and absorb specific wavelengths of visible light [23].

#### b) UV-Vis Spectral Study

**Fig. 2:**
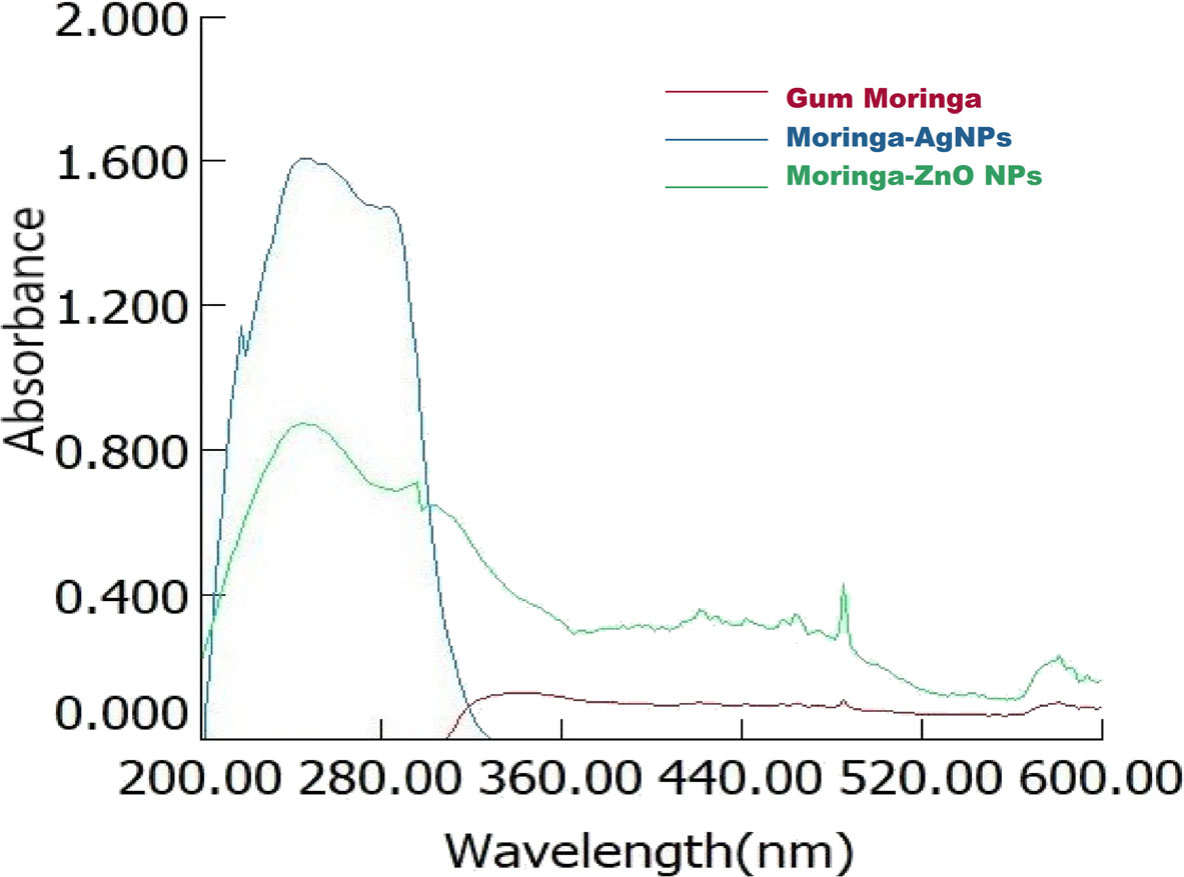
UV-Visible spectra of silver and ZnO nanoparticles

For characterization of metal nanoparticles, UV-Vis spectrophotometry is one of the most important tools. The absorption behaviour rises from “surface plasmon resonance”, which develops under electromagnetic field. Findings from high performance double beam *T80 + UV-Visible spectrophotometer-PG instruments* are presented in Fig. 2 reveals absorption pattern of GM, AgNPs and ZnO NPs synthesized through “green” method. It reveals that AgNPs developed comparatively with more efficiency than Zinc Oxide nanoparticles. Hydroxyl groups present in the gum Moringa reduced the silver and zinc. Synthesized nanoparticles exhibited absorption band in the range of 220–300 nm, a good Plasmon resonance band (PRB) of silver and Zinc NPs [24]. Absence of any peak at around 330nm and 550nm confirm the absence of nanoparticles aggregation [25]. No peak observed by gum sample as shown in Fig. 2.

#### c) Fourier-Transform Infrared Spectroscopy (FTIR) of synthesized NPs

**Fig. 3:**
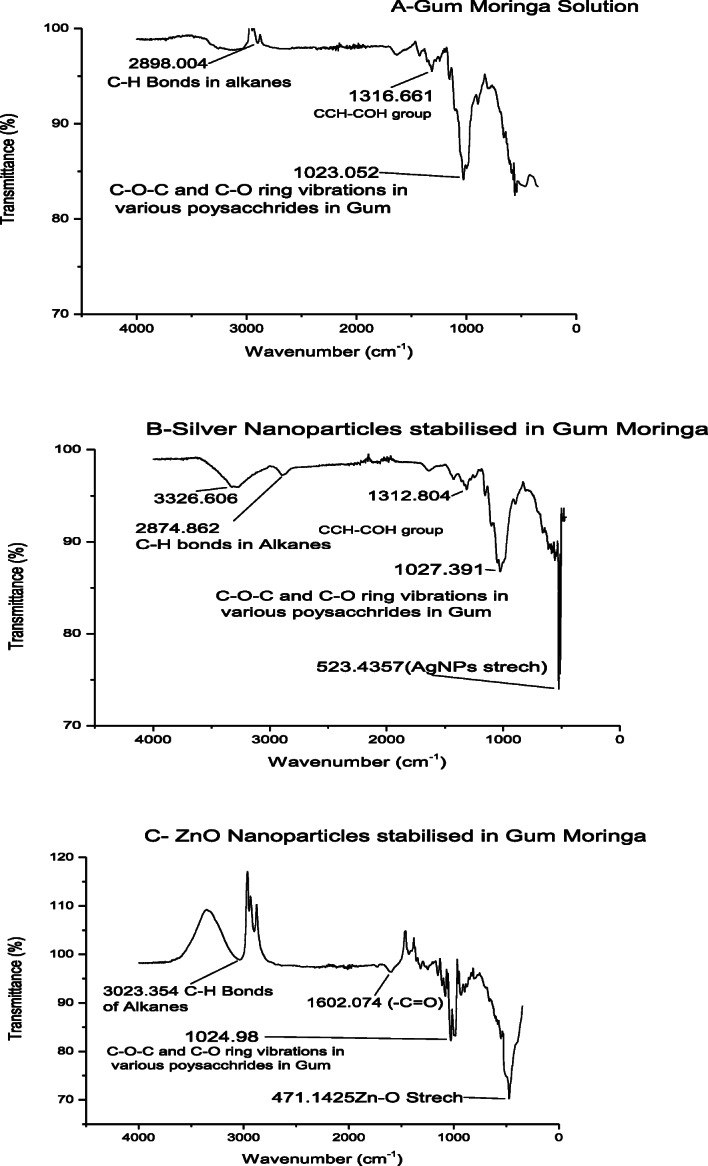
FTIR spectra of biosynthesized nanoparticles of Gum Moringa indicating AgNPs and ZnO NPs stretches

The Fourier transform infrared (FTIR) spectra *(IRAffinity-SHIZMADZU Japan)* revealed the stabilization of silver and zinc oxide nanoparticles by GM as shown in Fig. 3. Moringa-AgNPs and Moringa-ZnO NPs have shown characteristic metal nanoparticles bands at 523 cm − 1 and 471 cm − 1 respectively, which was absent in Gum Moringa. The band at 1602 cm − 1 is due to –C = O bending of acid group. Different types of polysaccharides present in gum and synthesized nanoparticles were expressed at 1023 cm − 1, 1027 cm − 1 and 1024 cm − 1. Variations in peak pattern of all three samples were due to process of reduction and stabilization of metals by flavonoids and reducing sugars in GM [26].

#### d) X-ray diffraction (XRD) and scanning electron microscope (SEM) analysis

To check the crystallinity of synthesized nanoparticles, X-rays diffraction analysis was performed using Rigaku *D/max-2400 diffracto-meter* with CuKα radiation.

**Fig. 4:**
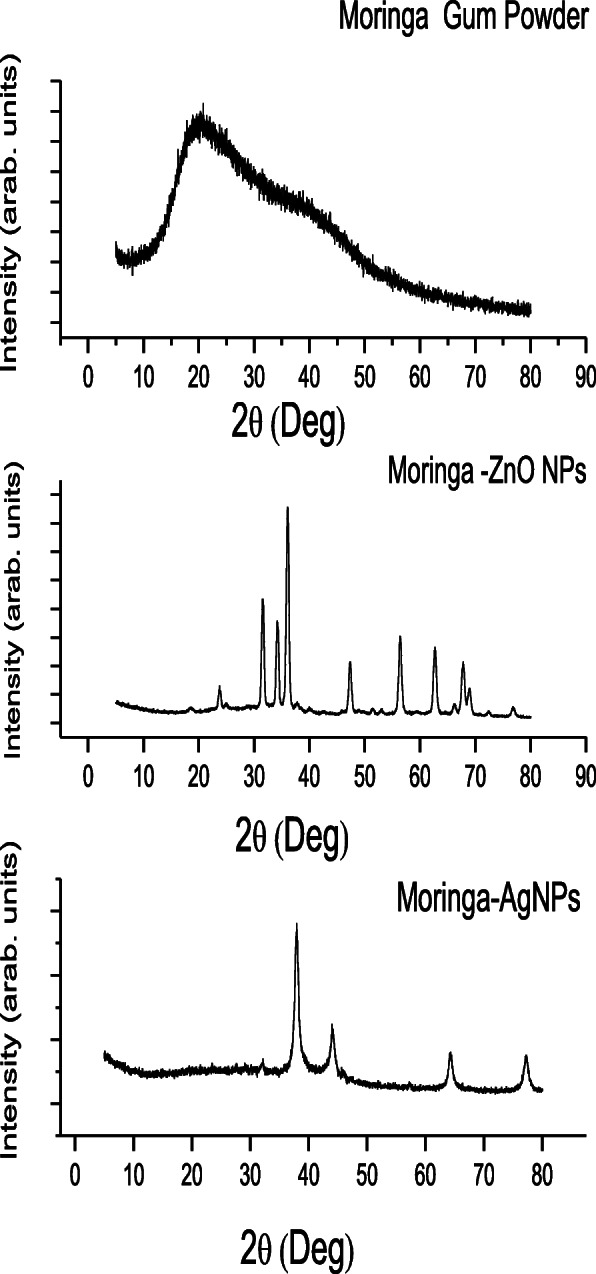
X-ray Diffractogram of GM Powder, Moringa-ZnO NPs and Moringa-AgNPs (From top to bottom)

Resulting diffractogram revealed excellent crystalline nature of developed gum based silver and zinc NPs as shown in Fig. 4. Gum powder amorphous nature was confirmed by diffractogram as it didn’t revealed any sharp diffraction pattern while AgNPs and ZnO NPs exhibited strong diffraction peaks.

**Fig. 5:**
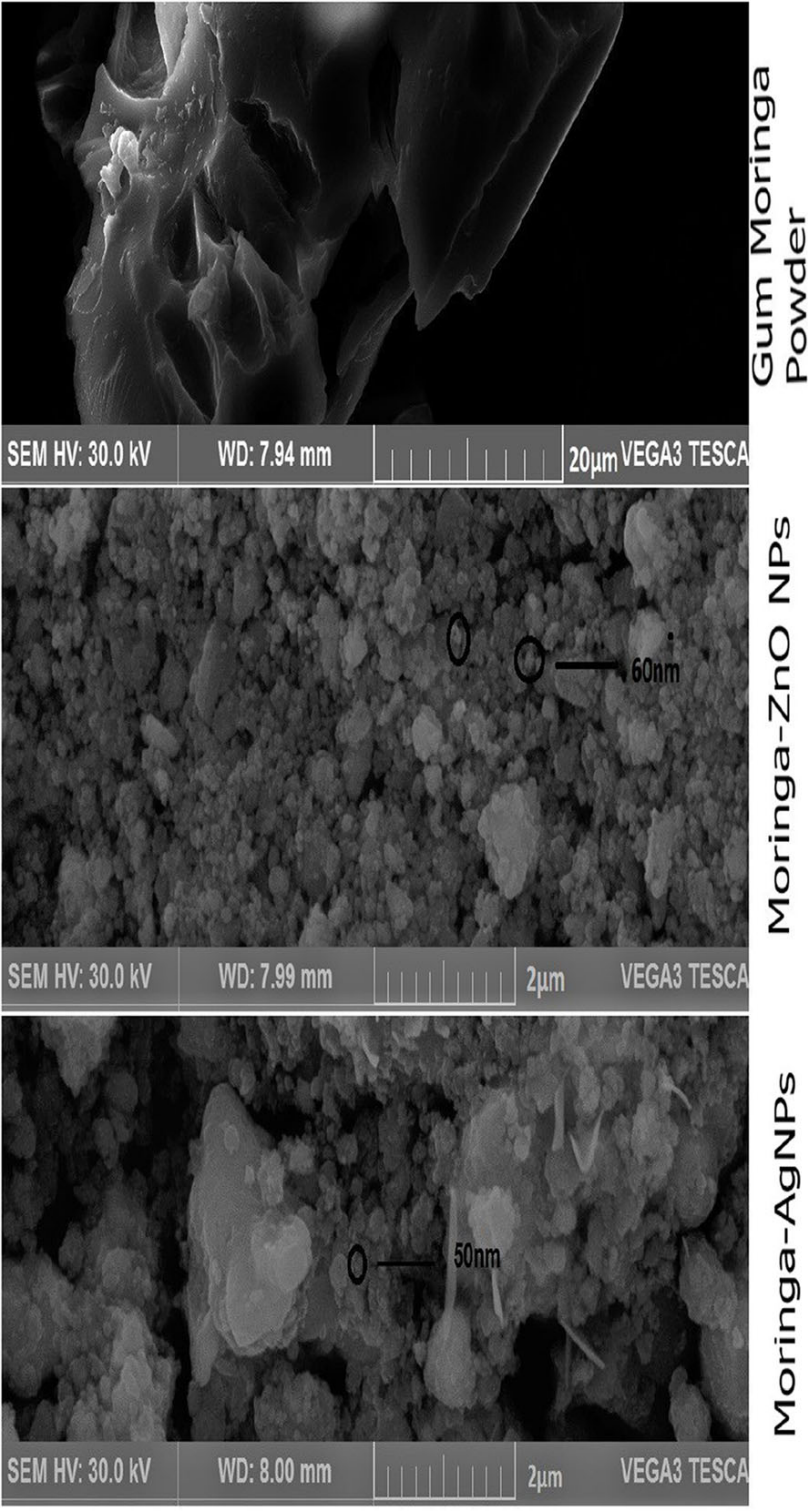
Scanning electron microscope (SEM) images of GM, Moringa-ZnO NPs and Moringa-AgNPs (From top to bottom)

The surface morphology of the gum powder, gum coated AgNPs and ZnO NPs were investigated using Scanning electron microscope (SEM) *TESCAN VEGA3-China* with an acceleration voltage of 30Kv and findings are presented in Fig. 5. It is clear from the image that the whole matrix is filled with small ZnO NPs and AgNPs indicating uniform distribution of nanoparticles. It is notable that finely grounded gum powder (purified) is with much higher size as compared to synthesized nanoparticles. Zinc oxide and silver nanoparticles were about size of 60nm and 50nm respectively. From nanomaterials point of view, this is an ideal size range [27].

### 2) Antibacterial activity against ***E.coli*** (gram negative), ***S. aureus*** (gram positive) and “super bug” MRSA

**Fig. 6:**
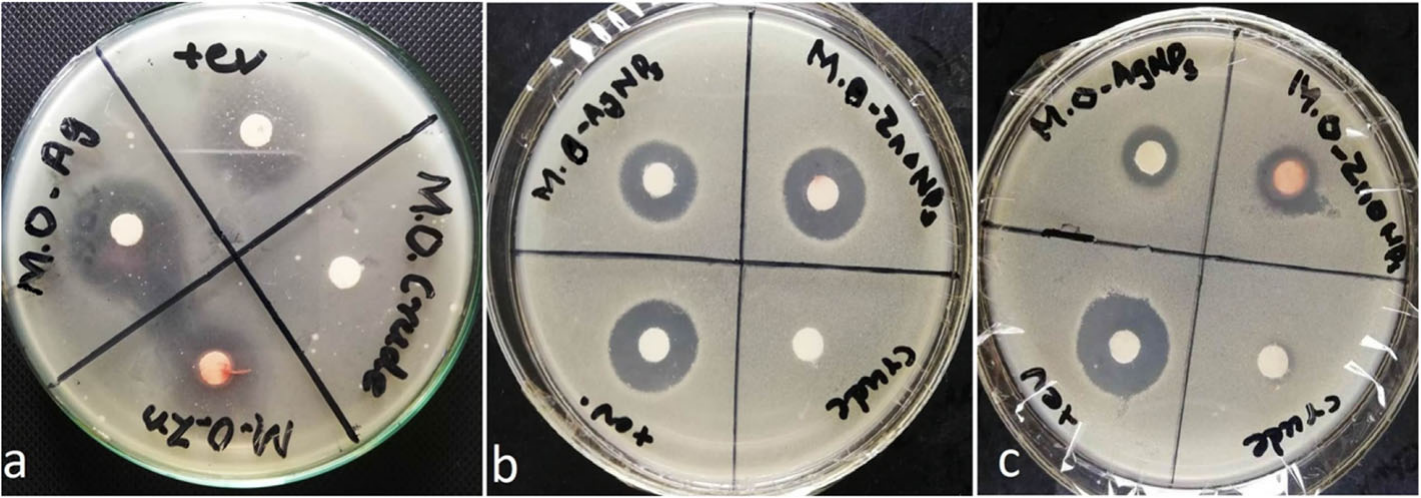
Gum based Ag and ZnO NPs antibacterial activity against *E.coli *(**a**)*, S. aureus *(**b**), and MRSA (**c**) along with positive control (i.e. Kanamycin for *E.coli*, *S.aureus* and Clindamycin for MRSA)

The antibacterial activity of AgNPs and ZnO NPs loaded GM polymers against *E.coli, S. aureus* and *methicillin-resistant Staphylococcus aureus* (MRSA) were investigated by widely used Kirby-Bauer antibiotic susceptibility test[28]. The susceptibility patterns for gum *Moringa oleifiera* (crude) solution, *Moringa olifiera* AgNPs, *Moringa oleifiera* ZnO NPs and Positive control (*kanamycin* for *E.coli* and *S.aureus* and *clindamycin* for MRSA) are shown in Fig. 6. To perform the test, 6mm filter paper discs were loaded with 20 µl of sample from the stock solution to make 30 µg/disc, 20 µg/disc, 10 µg/disc and 5 µg/disc final concentration of NPs via dilution method (Table 1). Each plate was loaded with positive control with concentration of 30 µg/disc. After that all plates were incubated at 37^o^C for 24 h[28].

**Table 1:** Zones of inhibition of synthesized biogenic nanoparticles against E.coli, S.aureus and MRSA

Samples	E. *coli* (Zone of inhibition, mm± SD)			*S.aureus* (Zone of inhibition, mm± SD)			*MRSA*(Zone of inhibition, mm± SD)		
Concentration	30 (µg/disc)	20 (µg/disc)	10 (µg/disc)	30 (µg/disc)	20 (µg/disc)	10 (µg/disc)	30 (µg/disc)	20 (µg/disc)	10 (µg/disc)
AgNPs	21 ± 02	15 ± 03	09 ± 02	20 ± 03	13 ± 02	08 ± 01	16 ± 03	11 ± 02	6 ± 03
ZnO NPs	22 ± 03	16 ± 03	10 ± 02	21 ± 02	14 ± 02	9 ± 03	17 ± 03	12 ± 01	6 ± 02
Crude	00	00	00	00	00	00	00	00	00
Positive control	24 ± 03	-	-	23 ± 03	-	-	24 ± 03	-	-

An excellent antibacterial activity was observed against both gram negative and gram positive bacteria *(E.coli and S.aureus)* while good activity was noted against *methicillin-resistant Staphylococcus aureus (MRSA)* as shown in Table 1; Fig. 6 (a, b, c). No zone of inhibition was observed at 5 µg/ml conc. for both testing samples and hence 10 µg conc. was considered as minimum inhibitory concentration (MIC). This indicates that GM-AgNPs and GM-ZnO NPs, due to their multiple mechanisms of action against *gram positive, gram negative* and *multi drug resistant bacteria* are good antibiotic agents and can be used further for biomedical applications.

## Discussion

Characteristic nature of Gum Moringa based metal NPs synthesized in this study were in line with previous reports of NPs characterization [29, 30].

Increasing popularity of green methods have resulted in different projects to synthesize Silver and ZnO NPs using bio based sources like fungus, bacteria, algae, protein, plants and others [31].

From decades, silver and zinc salts have been used to prevent the growth of different microbes from human. They were used in cuts, catheters, wounds and burns to protect them from infection[32]. Ag and ZnO NPs have been extensively studied for their activity against pathogenic bacteria like *Vibrio cholera, E. coli, Syphilis typhus*, *P. aeruginosa etc.*[33]. Thousands of patients died annually across the globe due to infection of resistant bacteria.

Due to emerging bacterial resistance to the available antibiotics, bacterial infections treatment has become alarming issue. There is dire need of new and potent bactericidal agents to cope with this problem. Metallic nanoparticles have shown promising results as antibacterial agents. NPs can targets bacterial cells via multiple routs i.e. by altering cell membrane permeability, protein activation, oxidative stress, enzyme activation and gene expression. Due to these unique properties, it becomes difficult for bacteria to develop resistance against NPs [33]. Our results added that that Gum Moringa based NPs not only worked against normal bacteria but also shown considerable activity against resistant bacteria (MRSA).

Metal NPs, due to their distinctive properties, have not only shown proven applicability in the field of medicine but also in catalysis, textile engineering, Nano biotechnology, bio-engineering sciences, optics, electronics and water treatment [22]. Novel NPs developed in this study can also be tested for said purposes.

However, there is further need of testing these NPs against fungus and other multi drug resistant bacteria. More characterization techniques can be applied to developed NPs. Detail mechanism of action of these NPs against microbes and resistant bacteria should be explored to unleash the underlying principles behind these important properties of NPs.

## Conclusions

*Moringa oleifera* Gum based Silver and Zinc Oxide nanoparticles were successfully prepared through eco-friendly, sustainable, economical and easy to use “green synthesis” process. Characterization with *UV-Visible*, FTIR, XRD, and SEM confirmed the chemical and physical properties of synthesized nanocomposites. A good antibacterial potential was shown by these nanomaterials against gram positive, gram negative and MDR bacterial strain. These finding encourages the use of these nanoparticles in field of nanomedicine and pharmaceuticals at industrial scale. Further in-depth study on these materials can open new horizon in the nanomedicine areas.

## Supplementary Information

**Additional file 1.**

**Additional file 2.**

**Additional file 3.**

## Data Availability

All data incorporated in article.
