# Supplementary figures and images for: Moringa oleifera gum based silver and zinc oxide nanoparticles: green synthesis, characterization and their antibacterial potential against MRSA
Source: Biomater Res. 2021 May 8;25:17. doi: 10.1186/s40824-021-00219-5 (PMC8106117; doi:10.1186/s40824-021-00219-5)

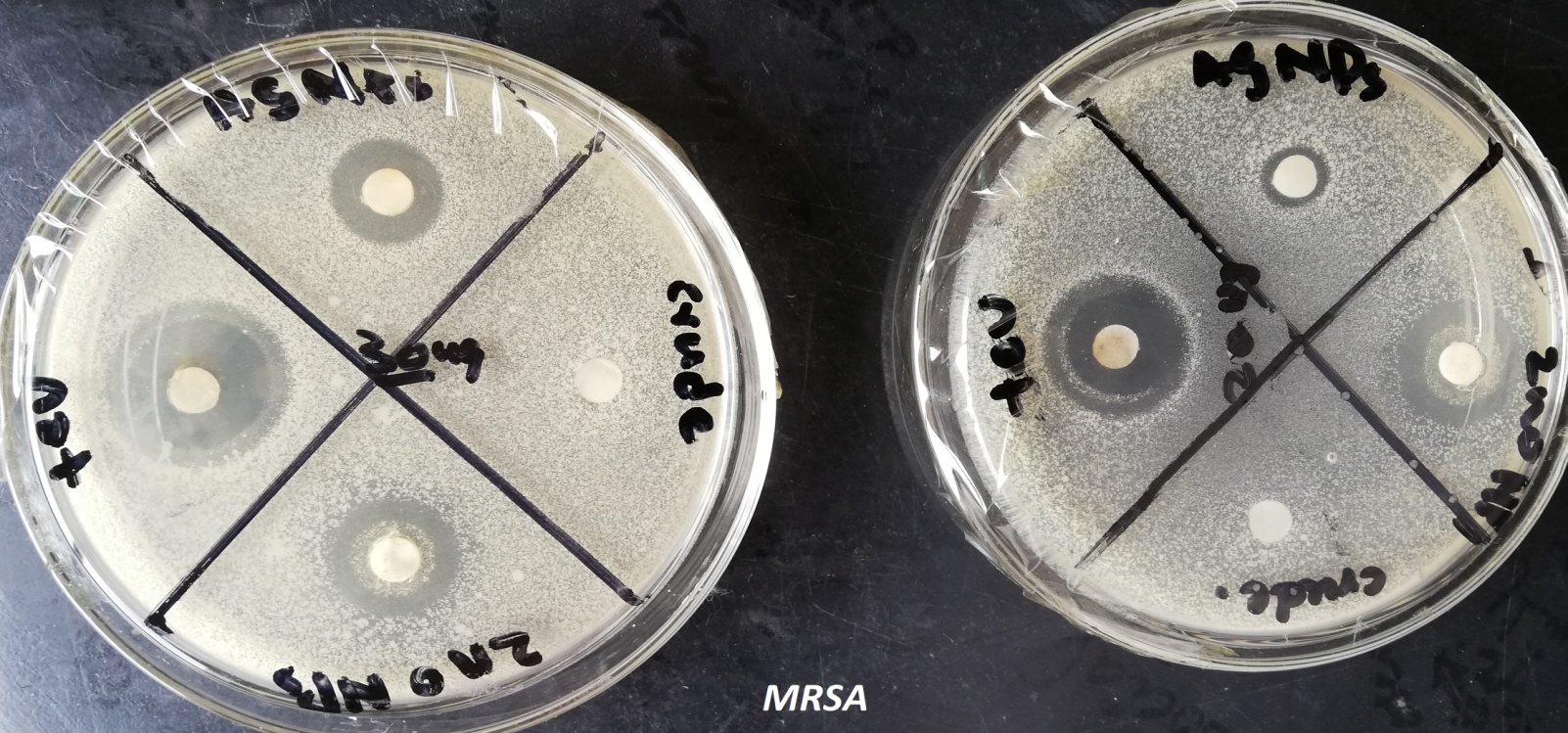


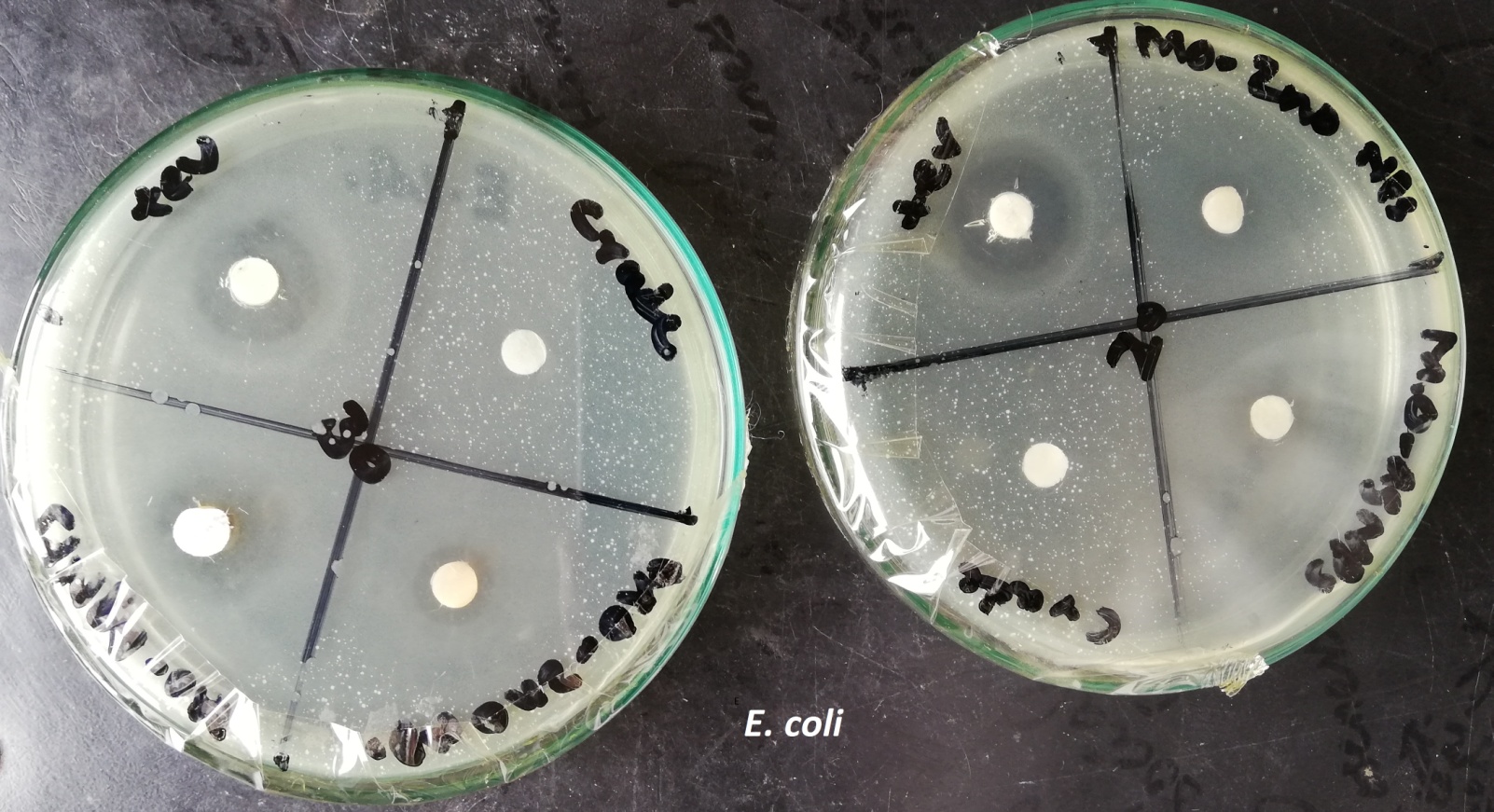


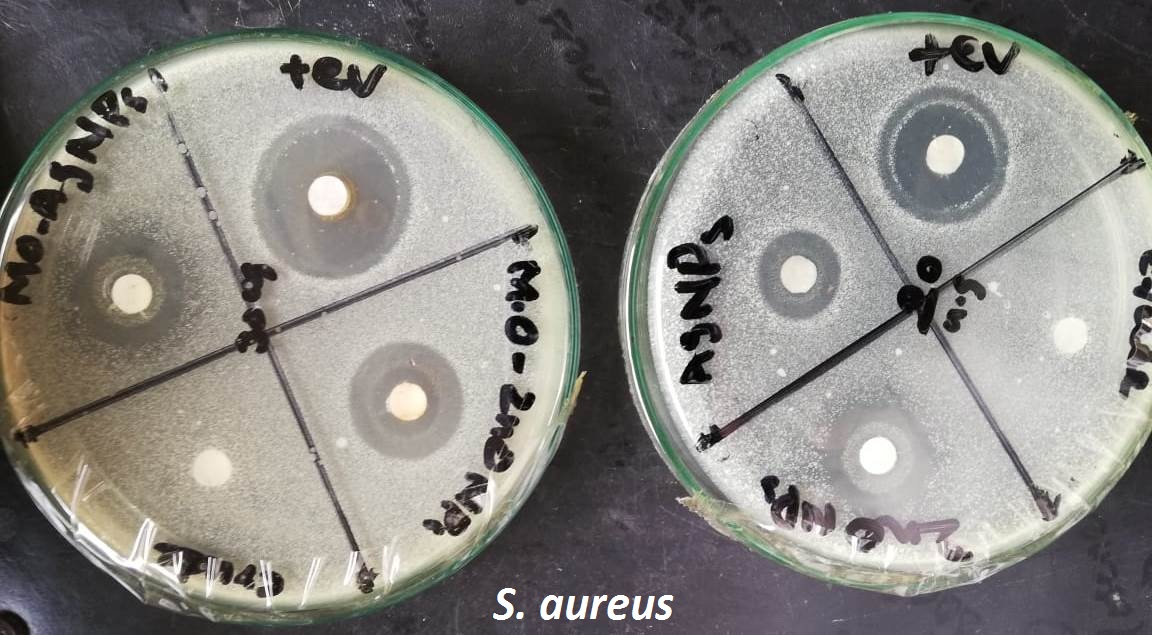

Supplement: Supplementary file 1 — Additional file 1. [file 40824_2021_219_MOESM1_ESM.docx]
